# *Addendum:* Hochreiter, B.; Pardo-Garcia, A.; Schmid, J.A. Fluorescent Proteins as Genetically Encoded FRET Biosensors in Life Sciences. *Sensors* 2015, *15*, 26281–26314

**DOI:** 10.3390/s151129182

**Published:** 2015-11-17

**Authors:** Bernhard Hochreiter, Alan Pardo-Garcia, Johannes A. Schmid

**Affiliations:** Institute for Vascular Biology and Thrombosis Research, Medical University Vienna, Schwarzspanierstraße 17, Vienna A-1090, Austria; E-Mails: bernhard.hochreiter@meduniwien.ac.at (B.H.); alanpardogarcia@gmail.com (A.P.-G.)

The authors wish to add an Acknowledgments section to their paper published in *Sensors* [1], doi:10.3390/s151026281, website: http://www.mdpi.com/1424-8220/15/10/26281.
